# A response to Rome: lessons from pre- and post-publication data-sharing in the *C. elegans *research community

**DOI:** 10.1186/1471-2164-11-708

**Published:** 2010-12-16

**Authors:** Matthew R Voell, Lily Farris, Edwin Levy, Emily Marden

**Affiliations:** 1 W. Maurice Young Centre for Applied Ethics, University of British Columbia, 227 - 6356 Agricultural Road, Vancouver, BC V6T 1Z2 Canada; 2 Child & Adolescent Mental Health & Addiction Services, An Agency of the Provincial Health Services Authority, Mental Health Building, Box 156, 4500 Oak Street, Vancouver, BC V6 H 3N1 Canada; 3 ISIS - A Research Centre at the Sauder School of Business, University of British Columbia, 2150 - 1055 W. Hasting Street, Vancouver, BC V6E 2E9 Canada

## Abstract

**Background:**

In recent years numerous studies have undertaken to measure the impact of patents, material transfer agreements, data-withholding and commercialization pressures on biomedical researchers. Of particular concern is the theory that such pressures may have negative effects on academic and other upstream researchers. In response to these concerns, commentators in some research communities have called for an increased level of access to, and sharing of, data and research materials. We have been studying how data and materials are shared in the community of researchers who use the nematode *Caenorhabditis elegans *(*C. elegans*) as a model organism for biological research. Specifically, we conducted a textual analysis of academic articles referencing *C. elegans*, reviewed *C. elegans *repository request lists, scanned patents that reference *C. elegans *and conducted a broad survey of *C. elegans *researchers. Of particular importance in our research was the role of the *C. elegans *Gene Knockout Consortium in the facilitation of sharing in this community.

**Results:**

Our research suggests that a culture of sharing exists within the *C. elegans *research community. Furthermore, our research provides insight into how this sharing operates and the role of the culture that underpins it.

**Conclusions:**

The greater scientific community is likely to benefit from understanding the factors that motivate *C. elegans *researchers to share. In this sense, our research is a 'response' to calls for a greater amount of sharing in other research communities, such as the mouse community, specifically, the call for increased investment and support of centralized resource sharing infrastructure, grant-based funding of data-sharing, clarity of third party recommendations regarding sharing, third party insistence of post-publication data sharing, a decrease in patenting and restrictive material transfer agreements, and increased attribution and reward.

## Background

In recent years numerous studies have undertaken to measure the impact of patents, material transfer agreements (MTAs), data-withholding and commercialization pressures on biological researchers [1,2]. At issue, and perhaps the impetus behind such studies, is the question of whether these factors have negative effects on the scope and quality of upstream research. In a classic paper on this subject, Heller and Eisenberg hypothesized that the proliferation of intellectual property rights in biomedical research had the potential to create an 'anticommons', whereby resources were prone to underuse because of the ability of multiple (patent) owners to exclude others from use of those resources [3]. Though scholars continue to debate the existence, scope and degree of that anticommons in the various fields of biology [1,2], some research communities have called for an increased level of access to, and sharing of, data and research materials as a result of these concerns [4,5].

As part of our work with the Intellectual Property and Policy Research Group (IPPRG) at the University of British Columbia, we have studied how data and materials are shared in the community of researchers who use the nematode *Caenorhabditis elegans *(*C. elegans*) as a model organism for biological research. Specifically, we conducted a textual analysis of academic articles referencing *C. elegans*, reviewed *C. elegans *repository request lists, scanned patents that reference *C. elegans *and conducted a broad survey of *C. elegans *researchers. Of particular importance in our research was the role of the *C. elegans *Gene Knockout Consortium (GKC) in the facilitation of sharing in this community. The GKC is a collaboration between three *C. elegans *labs, located in Canada, Japan, and the United States, whose mandate is to produce null alleles of all known genes in the *C. elegans *genome - creating knockout strains - and to share those strains with the public pre-publication [6].

Our purpose in writing this commentary is twofold. First, our study of sharing amongst *C. elegans *researchers provides insight into how sharing operates in this unique community and the role of the culture that underpins it. We believe that the greater scientific community can benefit from understanding these practices, as the *C. elegans *research community may stand as a model in this regard. In this sense, our research stands as a 'response' to calls for a greater amount of sharing in other research communities, specifically, to Schofield et al's 'Rome Agenda': a call for the creation of such a 'research commons' in the mouse research community [4]. Secondly, our study sheds light on the existence, or lack thereof, of drawbacks to upstream resource sharing, and explores whether an open system can still be productive and competitive.

## Methods

We adopted a four-pronged approach to measuring the sharing of data and materials within the *C. elegans *research community. Our belief was that this broad range of data would not only highlight objective measures of the existence of a research commons but also the subjective reasons behind such practices.

**Figure 1:**
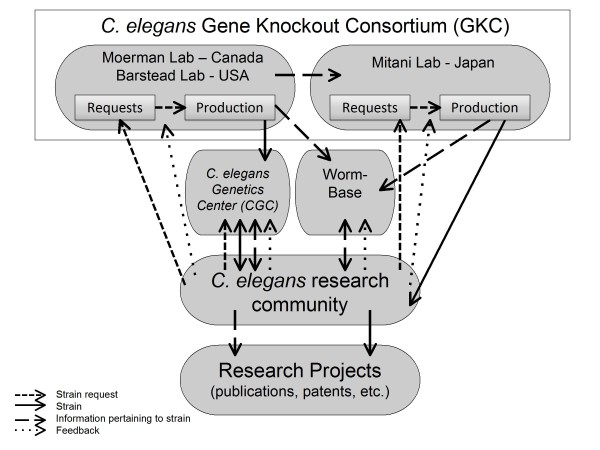
***C elegans *Gene Knockout Consortium**.

The first 'prong' of our research involved reviewing requests for *C. elegans *knockout strains from the Caenorhabditis Genetics Center (CGC). The CGC, housed at the University of Minnesota, collects, maintains and distributes stocks of *C. elegans*, and is the central repository for this research organism. We tracked references to strains produced by the labs that comprise the GKC and deposited in the CGC, in an attempt to measure the use of publicly available strains of *C. elegans *(See figure 1 for a diagram of the relationship between the GKC and CGC).

The second element of our approach was to quantify and examine references to GKC-produced *C. elegans *strains in academic peer-reviewed publications. We surmised that an important marker of the existence of a research commons was the attribution of pre-publication shared materials and thus sought to determine whether researchers who used the publicly shared strains attributed the creation of such strains to the CGC and GKC [7]. The literature search was done using WormBase, the central *C. elegans *database [8]; through which we identified articles that referenced the use of *C. elegans*. Articles were filtered to exclude those publications in which *C. elegans *was not a key component in the published research.

A related, third facet of our research was a search for patents that referred to *C. elegans*. We used the search term '*C. elegans*' in the United States Patent and Trademark Office patent search engine, collecting and reviewing references to *C. elegans *in both issued patents and pending patent applications. Our hypothesis was that this data would bear upon the question of whether sharing within a research community correlates in any manner with patenting.

The final, and most substantive dimension of our research was the development and dissemination of an online survey to scientific researchers who either work with *C. elegans *as a model research organism or work in an organization that uses *C. elegans *in their research. The survey was a web-based questionnaire that included 48 close-ended and 28 open-ended questions, asking researchers about their research experiences with *C. elegans*, their research practices, use of research materials, patenting practices and demographic information (See additional file 1 for the survey). After obtaining approval from the UBC Behavioral Research Ethics Board, our sample was drawn from registered members of WormBase, whose names appear in at least one *C. elegans *related publication included in WormBase. We excluded researchers who had retired, were no longer involved in laboratory work, or who were no longer working with *C. elegans *as a model organism. This group of excluded individuals was self-identified, as the first page of the survey invited only those researchers who were currently engaged in *C. elegans *research to complete the survey.

We used a mixed methods approach to sampling researchers, and sent out five waves of survey invitations according to our list of 652 registered *C. elegans *researchers. We followed this invitation to action with a simple random sample of 400 respondents who received an additional email encouraging them to respond to the survey. We also used a snow-ball sampling method to expand our pool of potential respondents to researchers in all aspects of *C. elegans *research, requesting any *C. elegans *researcher who received the survey request to forward the link to other individuals who were involved in any aspect of *C. elegans *related research. Ultimately we received 680 responses from our sample of *C. elegans *researchers.

## Results

Among survey respondents, 410 (60.3%) reported having deposited knockout strains into the CGC. As of December 2009, 3,883 of the 12,171 (31.9%) strains deposited in the CGC were deposited by the three labs that comprise the GKC. Survey respondents reported receiving CGC strains (94.4% of respondents) and GKC strains specifically (76.9% of respondents). Furthermore, survey respondents often commented on the significance of the publicly available strains to their work, especially those working in small labs: "I have a very small lab and don't have the resources to make my own knockouts."

Since the establishment of the GKC in 2001, over one thousand academic references have been made to strains produced by the Canadian and American components of the consortium, the breadth of publications ranging from *Nature *to the *Worm Breeder's Gazette*. The quantity of publications referencing the use of GKC strains has steadily increased since its inception, from 22 in 2001 to 212 in 2009 [9]. Of those articles that reference using these strains, 95 report using more than one GKC produced strain.

The *C. elegans *researchers who responded to our survey indicate that for the most part they did not use formal intellectual property mechanisms (e.g. MTAs) when acquiring and distributing data and research materials: 453 (66.6%) and 544 (79.7%), respectively. Of particular significance is the fact that the American and Canadian components of the GKC deposit knockout strains in the CGC without requiring requestors to complete an MTA for use of the strains. While the lab in Japan does require an MTA for use of the strains they produce, researchers did not once mention, in their survey responses, that MTAs impeded their research. In addition to the informality with which they share materials, 177 (26.0%) of survey respondents report being listed as an inventor on a patent application. While this is a fairly high percentage for a 'basic research' community, it may be significant that many respondents reported that when filing for patents they did so at the request of their funding body. The results of the keyword-based patent search determined that the number of *C. elegans *related patents granted in the United States has increased steadily from 1976 to 1999, and since then has held steady at about 250-300 granted patents a year. *C. elegans *were usually referenced as prior art to the claimed invention, most often used in early stages of product exploration and/or development.

A number of survey questions asked *C. elegans *researchers about their perceptions toward data-sharing and research materials (See additional file 1 for sample survey questions). The majority of worm researchers surveyed report sharing data and research pre- and post-publication, regardless of the requirements of their funding bodies. In addition, most respondents reported the belief that *C. elegans *samples should be shared freely: 518 (76.2%) agreed that one should not restrict access or use of scientific data; 616 (90.6%) reported that they encourage their colleagues to share data and research materials. When respondents were asked about their views on commercialization, they were much more divided in their response. Responses were roughly evenly split between those who agreed that granting exclusivity in exchange for disclosure through intellectual property rights preserves incentives to innovate, and those that disagreed. Most respondents did agree however that focusing on research with potential commercial outputs may impair the free exchange of materials. Finally, roughly a third of respondents reported that they would create a private-start up company should they have the opportunity.

## Discussion

Our analysis of this diverse data set suggests that a scientific commons, or culture of sharing, exists within the *C. elegans *community. In this sense, the data speaks to the viability of recent calls for increased sharing in other research communities and provides insight into what practices may successfully facilitate such sharing [4].

Firstly, our research demonstrates the strength gained from openly available, publicly funded infrastructure. Within the worm community such infrastructure centralizes the sites of resource production (GKC), housing of experimental resources (CGC) and online research material (WormBase). Centralized funding of data production and repository projects creates opportunities for smaller scale researchers to focus their work on hypothesis driven experiments rather than laborious production, as well as increases the productivity of such research. It is simply more cost-effective to create a knockout once, and share it, than the parallel production of identical knockouts. This practice also allows better data comparison across *C. elegans *labs, given the standardization of knockout strains. Furthermore, if granting organizations are in the business of funding multiple hypothesis driven research projects, it may be more cost-effective for these bodies themselves to fund projects that centrally produce research materials from which other researchers can draw.

While most *C. elegans *researchers reported sharing absent explicit directions to do so by funding bodies, the practice of data-sharing varies among researchers and research communities. Some researchers reported sharing data only when explicitly asked to do so, while others reported regular contributions to central repositories. It may be that clear recommendations for sharing by funding agencies would 'normalize' the process of sharing, however, within the worm community such directives seem to be unnecessary. That said, given the lack of a tradition of sharing within other research communities, it may be important that funding bodies give such recommendations and direction to facilitate sharing. Similarly, our study of sharing in the worm community suggests that while pre- and post-publication of data and materials does occur in the worm community, it is not because of the insistence of third parties (scientific journals, grant reviewers, and funding bodies). Rather, we hypothesize that sharing occurs as a result of the ethos found within the community and first espoused by Sydney Brenner and John Sulston [10].

From our study, it is clear that while *C. elegans *strains are widely used as research tools, the large amount of sharing does not preclude patenting, in spite of the stated preference by prominent members of the research community for the public domain [10]. It is worth noting however that there is very little patenting of the organism itself, due perhaps to the lack of *direct *market applicability of the results of *C. elegans *research. In addition, the fact that specific knockouts or *C. elegans *DNA sequences are generally not patented as research tools may facilitate sharing within this research community. Certainly, post-publication dissemination and MTA-free sharing may be more likely where researchers are not overly concerned with protecting proprietary interests granted by patents.

Our survey data suggests that WormBase, the central resource for data, metadata and related computational tools for worm researchers, plays a significant role in facilitating the *C. elegans *research commons. The WormBase management and curatorial team set standards for data and metadata and implement new standards as they develop. The WormBase curatorial team also invests in computational tools and reviews data before it appears online. As a result of these efforts, issues related to data standardization are addressed by the community. Given the success of WormBase in the standardization of tools and information for *C. elegans *researchers, other research communities would be wise to adopt such a model if databases do not exist, and invest time and resources into them if they do.

Like other research communities, members of the *C. elegans *community are requested to recognize the contribution of their colleagues when using pre-publication shared strains, data and materials. In the worm community, researchers are generally asked to acknowledge groups, such as GKC and CGC, when using shared materials, and are not required to add additional authorship unless the researcher has made a major contribution to their work. Even if a researcher does not explicitly acknowledge the source of the strains employed in her research, the nomenclature of strains identifies those originating from the GKC. Thus, the phenomenon of pre-publication sharing is visible to all.

## Conclusions

Our research suggests that a culture of sharing exists within the *C. elegans *research community. Such a practice of sharing is informative for the establishment of a creative commons within other research communities. Specifically, our research speaks to the strength of Schofield et al's call for increased investment and support of centralized resource sharing infrastructure, grant-based funding of data-sharing, clarity of third party recommendations regarding sharing, third party insistence of post-publication data sharing, a decrease in patenting and restrictive MTAs and increased attribution and reward.

## List of abbreviations used

***C. elegans*: ***Caenorhabditis elegans***; CGC: **Caenorhabditis Genetics Center; **GKC: ***C. elegans *Gene Knockout Consortium; **IPPRG: **Intellectual Property and Policy Research Group; **MTA: **material transfer agreement;

## Competing interests

The authors declare that they have no competing interests.

## Authors' contributions

MRV drafted the manuscript. LF helped design the study, performed the survey, literature review, patent search and participated in drafting the manuscript. EL and EM helped conceive and design the study and draft the survey and manuscript. MRV and LF should be considered as joint first authors. All authors read and approved the final manuscript.

## Authors' information

The authors are all members of the Intellectual Property and Policy Research Group (IPPRG), a collaboration of interdisciplinary researchers interested in the convergence of science, technology, innovation and translation. For more information about the IPPRG please visit: http://ipprg.wordpress.com.

## Supplementary Material

Additional file 1 **C elegans Researcher Survey**. Additional file 1 contains the online survey, which was completed by all survey respondents. Click here for file
