# Supplementary material for: A response to Rome: lessons from pre- and post-publication data-sharing in the C. elegans research community
Source: BMC Genomics. 2010 Dec 16;11:708. doi: 10.1186/1471-2164-11-708 (PMC3019234; doi:10.1186/1471-2164-11-708)
Supplement: Additional file 1 — C elegans Researcher Survey. Additional file 1 contains the online survey, which was completed by all survey respondents. [file 1471-2164-11-708-S1.PDF]

Dear Participant,

My name is Ed Levy and I am an adjunct professor at the W. Maurice Young Centre for Applied Ethics at the University of British Columbia working in collaboration with my co-investigator Lily Farris and the members of the Intellectual Property Policy Research Group on Genome Canada funded GE3LS (genomics, ethics, environment, economics, law and society) research.

Study Procedure:

We are contacting you to participate in this study of researchers who use *C. elegans* as a model organism. This survey will provide key information about your current use and exchange of research materials. You are invited to participate in this survey if you are 19 years of age or older and engage in any part of a research process which uses *C. elegans* (nematodes) as the model organism. Your participation is essential if the survey results are to be accurate and representative.

This online survey (www.wormsurvey.com) will take approximately 10 minutes to complete. Please circulate this message and survey to all members of your research team including graduate students, technicians, and research assistants who conduct any *C. elegans* related research. If you find any comments, issues, or challenges with the construction or use of the survey please contact us.

Purpose:

We are researching how publicly available data is accessed and how this process informs scientific advancement. Our survey asks researchers about their scientific resource use, sharing of information, and handling of information with proprietary potential. The results from this survey will be used to better understand the relationship between the researcher's use and exchange of information and their scientific innovation.

Potential Risks:

There are no potential risks to participation. You may experience minimal levels of discomfort when discussing challenging experiences at work.

Potential Benefits:

There are no direct benefits to participation in this survey. The results from this survey will inform the on-going public, scientific, and policy dialogue on the impact of basic science research. This survey gives you an opportunity to provide feedback on the current systems of data exchange and provide suggestions on how science infrastructure is best

[illegible] created, developed, maintained and funded.

Confidentiality:

All information we collect in this voluntary survey will be kept strictly confidential. You are free to skip questions that you prefer not to answer or to terminate your participation in the survey at any time. This data will in no way be personalized and will only be referenced in aggregate form. We recommend that you print a copy of this information page for your own records.

Consent:

Your consent to be involved in this study is assumed when you submit this survey. Refusal to participate or withdrawal from this survey will have no effect on your employment or involvement in any *C. elegans* related organizations.

Your rights as a research subject:

You are welcome to contact the Research Subject Information Line in the UBC Office of Research Services (604-822-8598 or RSIL@ors.ubc.ca) if you have any concerns about your treatment or rights as a research subject.

Results from this survey will be summarized and made available to you upon request. If you would like to learn more about our research please visit http://gels.ethics.ubc.ca:8213/ge3ls-arch/intellectual-property/the-ge3ls-c-elegans-gene-knockout-consortium

If you have any questions or comments please contact Lily Farris (lfarris@interchange.ubc.ca, cell: 604-614-5745) or me at the University of British Columbia W. Maurice Young Centre for Applied Ethics.

Thank you for your participation.

Sincerely,

Edwin Levy, Ph.D.

Principal Investigator

Intellectual Property and Policy Research Group (IPPRG)
The W. Maurice Young Centre for Applied Ethics
The University of British Columbia, 227-6356 Agricultural Road Vancouver, BC, V6T 1Z2
General Office: 604-822-8625; Fax: 604-822-8627; elevy@telus.net

C. elegans Researcher Survey

2. Background Information

We would like to ask you about your scientific background.

1. What fields of science are you primarily working in?
If you are retired or on leave, in what area of science did you work?

Check all that apply:

- ☐ Biological sciences
- ☐ Bioinformatics
- ☐ Genetics
- ☐ Genomics
- ☐ Biochemistry
- ☐ Medical/health sciences
- ☐ Agricultural sciences
- ☐ Other life sciences
- ☐ Physical sciences
- ☐ Chemistry
- ☐ Engineering
- ☐ Earth Sciences
- ☐ Math and computer sciences
- ☐ Science history, ethics, or philosophy
- ☐ Science education or administration
- ☐ Science publishing or media
- ☐ Other social, behavioural, or economic sciences
- ☐ Other (please specify) __________

2. How would you describe your job title or project role?

Check all that apply:

- ☐ Academic teaching
- ☐ Academic research
- ☐ Professor, full
- ☐ Professor, associate
- ☐ Professor, assistant

- Undergraduate student
- Undergraduate student (Co-op, work study, directed studies)
- Graduate student
- Post-doctoral fellow
- Principal Investigator
- Technician
- Technician, supervisory role
- Research Assistant
- Research Associate
- Scientific research (non-academic)
- Academic administration (e.g., Dean, Department Head)
- Administration/Management (University/corporate/government)
- Development (or Finance)
- Funding
- Medical services
- Product/process engineering
- Intellectual property management
- Retired/on leave
- Other (please specify)

3. What model organisms do you currently work with?
This may include model organisms that are used by your laboratory or organization, but that you aren't working with directly.

Check all that apply:

- Bacteria
- Cat (*Felis cattus*)
- Chicken (*Gallus gallus domesticus*)
- Cnidarian (*Hydra* genus)
- Dog (*Canis lupus familiaris*)
- Frog (*Xenopus laevis*)
- Fruit fly (*Drosophila melanogaster*)
- Guinea pig (*Cavia porcellus*)
- Human (*Homo sapiens*), including all material of human origin
- Mouse (*Mus musculus*)
- Nematode (*Caenorhabditis elegans*) and other types of worms
- Purple sea urchin (*Strongylocentrotus purpuratus*)
- Rat (*Rattus norvegicus*)
- Sea slug (*Aplysia californica*)
- Sea squirt (*Ciona intestinalis*)
- Sea urchin (*Arbacia punctulata*)
- Yeast (*Saccharomyces cerevisiae*)
- Zebrafish (*Danio reiro*)

☐ Other (please specify) Common name: __________

Latin name: __________

What model organisms have you worked with in the past, that you are not currently working with?

____

Next >>

C. elegans Researcher Survey

3. Data Sharing and Intellectual Property

The following questions address your experience with patenting and the use of scientific resources.

4. Have you ever been listed as an inventor on a patent (provisional patent, patent application, or issued patent)?

○ Yes ○ No

If you answered **Yes**:

How many patents have you been listed as an inventor on? ☐

Which statement(s) below best describes your most recent experience as an inventor on a patent application?

- ☐ The organization which funded my research encouraged me to seek a patent when possible.
- ☐ The organization which funded my research required me to seek a patent when possible.
- ☐ I was required to work with a technology transfer office.
- ☐ I chose to, but was not required to, work with a technology transfer office.
- ☐ Other

If you selected "Other", please specify: ☐

If you answered **No**:

Have you ever had a discovery that appeared to be patentable but that you chose not to patent?

○ Yes ○ No

If yes, why? ☐

5. Have you ever acquired data or research materials from another researcher, lab, or organization?

- ☐ Yes, concerning published data or research materials.
- ☐ Yes, concerning unpublished data or research methods.
- ☐ No (skip to question #7)

6. When you most recently acquired data or materials from another researcher, what

intellectual property mechanism was used to acquire the data or information?

- ☐ Informal (no official agreement)
- ☐ Material Transfer Agreement (MTA)
- ☐ License (exclusive)
- ☐ License (non-exclusive)
- ☐ Sponsored Research Agreement (SRA)
- ☐ Confidentiality agreement
- ☐ Memorandum of Understanding (MOU)
- ☐ General Public License (GPL, Open Source Software)
- ☐ Other (*please specify*) ________

7. During the past two years, have other scientists asked you to provide information, data, or research materials for them?

- ☐ Yes, concerning published data or research materials.
- ☐ Yes, concering unpublished data or research methods.
- ☐ No (skip to question #9)

8. When you most recently gave data or materials to another researcher, what intellectual property mechanism was used to facilitate the transfer of data or information?

- ☐ Informal (no official agreement)
- ☐ Material Transfer Agreement (MTA)
- ☐ License (exclusive)
- ☐ License (non-exclusive)
- ☐ Sponsored Research Agreement (SRA)
- ☐ Confidentiality agreement
- ☐ Memorandum of Understanding (MOU)
- ☐ General Public License (GPL, Open Source Software)
- ☐ Other

Next >>

C. elegans Researcher Survey

4. *C. elegans* resources

These questions ask about resources specific to the *C. elegans* research community.

9. How often have you retrieved data or information from Wormbase?

Wormbase is a repository of mapping, sequencing, and phenotypic information about the nematode *C. elegans*.

- Never
- Daily
- Weekly
- Monthly
- Annually

10. Have you ever received strains from the CGC (*Caenorhabditis* Genetics Center)?

The *Caenorhabditis* Genetics Center is a repository for *C. elegans* information and strains located at the University of Minnesota.

- Yes
- No

If you answered **Yes**:

How many times in the past year have you received strains from the CGC?

11. Have you ever contributed strains to the CGC (*Caenorhabditis* Genetics Center)?

- Yes
- No

If you answered **Yes**:

How many times in the last three years have you contributed strains to the CGC?

Next >>

C. elegans Researcher Survey

4. *C. elegans* resources

These questions ask about resources specific to the *C. elegans* research community.

12. Have you ever received alleles (or other materials) from the *C. elegans* Gene Knockout Consortium or from the CGC (*Caenorhabditis* Genetics Center) which originated in the *C. elegans* Gene Knockout Consortium (i.e., any 'gk' or 'ok' allele)?

Select an option:

If you selected "No, for another reason" *above, please specify the reason:*

If you selected "Yes" *above, please indicate how many alleles you have received:*

13. Have you found cases where a strain reported to be homozygous carried a wild type copy of the target gene?

- Yes *If you answered yes, how many times did this occur?*
- No
- I have never received consortium strains

Your comments on this topic (if any):

14. Have any of the materials you received from the *C. elegans* Gene Knockout Consortium been used in any stage of commercial development?

- Yes
- No
- Don't know
- I have never received materials from the GKC

Your comments on this topic (if any):

15. Have you ever received strains where a variant phenotype was not readily apparent?

- Yes
- No
- I have never received consortium strains

Your comments on this topic (if any):

Next >>

C. elegans Researcher Survey

4. *C. elegans* resources

These questions are about resources specific to the *C. elegans* research community.

16. In your publications, have you cited strains originating from the *C. elegans* Gene Knockout Consortium?

- Yes
- No
- I don't have any publications

If you answered **No**: Why not?

Please elaborate:

17. When you received consortium strains from the CGC, did you outcross them to wild type to clean up the background?

- Yes *If you answered* yes*:* On average, how many generations do you out-cross to?
- No *If you answered* no*:* Why not?
- I have never received consortium strains

18. Have you done tests to confirm the genotype of the strain?

- Yes
- No

If you answered **Yes**:

Did you run a PCR with published primer sequence to confirm genotype?

19. If you had any issues or feedback regarding materials originating from the *C. elegans* Gene Knockout Consortium, did you contact the consortium?

- Yes
- No

Your comments on this topic (if any):

20. Have you done tests to confirm that the protein of interest was absent or reduced?

- Yes
- No

Your comments on this topic (if any):

Next >>

C. elegans Researcher Survey

5. Scientific Resources

This question deals with your use of resources available from the *C. elegans* Gene Knockout Consortium.

21. What is your level of agreement with each of the following statements?

	Agree strongly	Agree	Disagree	Disagree strongly
1. I use knockouts from the *C. elegans* Gene Knockout Consortium to explore new research opportunities.	○	○	○	○
2. The alleles I receive from the *C. elegans* Gene Knockout Consortium are important to my work.	○	○	○	○
3. My lab would function differently if the *C. elegans* Gene Knockout Consortium was not available.	○	○	○	○
4. Using strains from the *C. elegans* Gene Knockout Consortium was a key part of my data interpretation.	○	○	○	○
5. I would use the *C. elegans* Gene Knockout Consortium again for future research.	○	○	○	○
6. I would recommend the *C. elegans* Gene Knockout Consortium to others.	○	○	○	○

Your comments on this topic, if any:

Next >>

C. elegans Researcher Survey

6. Scientific Resources

This question deals with your use of scientific resources available to the *C. elegans* research community.

22. What are your opinions or views on the following technological practices?

	Agree strongly	Agree	Disagree	Disagree strongly
1. Samples should be shared freely between research organizations.	○	○	○	○
2. Data you don't plan on publishing should be shared between research organizations.	○	○	○	○
3. Source code, or the set of instructions behind a particular computer program, should be given away freely.	○	○	○	○
4. No one should restrict access or use of scientific data.	○	○	○	○
5. I encourage my colleagues to exchange research materials.	○	○	○	○
6. Granting exclusivity in exchange for disclosure through Intellectual Property rights preserves incentives to innovate.	○	○	○	○
7. Focusing research on potential commercial outputs may impair the free exchange of materials.	○	○	○	○
8. I think patent protection should be sought whenever possible.	○	○	○	○
9. I would create a private start-up company if I had the opportunity.	○	○	○	○

Your comments on this topic, if any:

Next >>

C. elegans Researcher Survey

7. Research Dissemination

Here, we would like to ask you about your dissemination of research results.

23. What are the last two peer-reviewed journals you published in?

Journal name

Most recent publication: [ ] Year: [ ]

If you selected "Other", please specify: [ ]

Journal name

Second most-recent publication: [ ] Year: [ ]

If you selected "Other", please specify: [ ]

24. What were the last three scientific conferences or meetings you attended?

Conference (or meeting) 1: [ ] Year [ ]

Conference (or meeting) 2: [ ] Year [ ]

Conference (or meeting) 3: [ ] Year [ ]

☐ I have attended no scientific conferences or meetings

Next >>

C. elegans Researcher Survey

8. Educational Information

These are more general questions about your background.

25. In which type of institution are you mainly employed?

Check all that apply:

- ☐ University research institute
- ☐ Academic hospital
- ☐ Private business or industry
- ☐ Private non-profit organization
- ☐ Government organization
- ☐ Other

If you selected "Other", *please specify:* __________

Have you ever been employed in any other types of institutions? (*Check all that apply*.)

- ☐ University research institute
- ☐ Academic hospital
- ☐ Private business or industry
- ☐ Private non-profit organization
- ☐ Government organization
- ☐ Other

If you selected "Other", *please specify:* __________

26. How many years of experience do you have in your current area of specialization?

Number of years: _____

27. What degrees have you completed?

Check all that apply:

- [ ] Associate degree (e.g., AA)
- [ ] Foundation degree (e.g., FdA)
- [ ] Bachelor's degree (e.g., BA or BS)
- [ ] Master's degree (e.g., MA or MS)
- [ ] Doctoral degree (e.g., Ph.D)
- [ ] Medical Doctor (e.g., MD)
- [ ] Other

If you selected "Other", *please specify:*

28. What are your top two sources of research funding?

Primary source of funding:

Secondary source of funding:

29. In which country have you primarily been trained?

Country

Select a country:

30. In which country were you born?

Country

Select a country:

31. What is your gender?

( ) Female ( ) Male

32. In what year were you born?

Year

Select a year:

Done >>

C. elegans Researcher Survey

9. Follow-up

Thank you for your participation!

You're now finished with the survey.

Would you be willing to be contacted for a follow-up interview by telephone, in which we will ask you additional questions about your research experiences and intellectual property practices?

(A random sample of thirty respondents will be contacted for follow-up interviews.)

- Yes
- No

Submit >>
